# First Molecular Identification of *Calicophoron daubneyi* (Dinnik, 1962) and *Paramphistomum leydeni* (Nasmark, 1937) in Wild Ruminants from Romania

**DOI:** 10.3390/vetsci10100603

**Published:** 2023-10-03

**Authors:** Sorin Morariu, Cătălin Bogdan Sîrbu, Adrienn Gréta Tóth, Gheorghe Dărăbuș, Ion Oprescu, Narcisa Mederle, Marius Stelian Ilie, Mirela Imre, Beatrice Ana-Maria Sîrbu, Norbert Solymosi, Tiana Florea, Kalman Imre

**Affiliations:** 1 Department of Parasitology and Parasitic Diseases, University of Life Sciences King Mihai I, 300645 Timisoara, Romania; sorin.morariu@fmvt.ro (S.M.); gheorghe.darabus@fmvt.ro (G.D.); ioan.oprescu@fmvt.ro (I.O.); narcisamederle@usvt.ro (N.M.); marius.ilie@fmvt.ro (M.S.I.); mirela.imre@usvt.ro (M.I.); beatrice.jitea@usvt.ro (B.A.-M.S.); 2 Department Centre for Bioinformatics, University of Veterinary Medicine Budapest, Budapest István u. 2, 1078 Budapest, Hungary; tothadrienngreta@gmail.com (A.G.T.); solymosi.norbert@univet.hu (N.S.); 3 Food Safety Department, University of Life Sciences King Mihai I, 300645 Timisoara, Romania; kalmanimre@usvt.ro

**Keywords:** roe deer, western Romania, PCR, rumen flukes

## Abstract

**Simple Summary:**

Rumen flukes are geographically widespread trematodes affecting wild and domestic ruminants. Severe diarrhoea and weight loss are the major clinical signs, and the disease might be fatal in severely infested individuals. Fifty-two pre-stomachs (rumen and reticulum) obtained from roe deer (*Capreolus capreolus*) that were hunted on 14 hunting grounds from Timiș and Arad counties were examined for the presence of paramphistomes. Three (9.09%) out of 33 samples were positive in Timiș County, and one (5.26%) out of 19 samples was positive in Arad County, the main identified species being *C. daubneyi* and *P. leydeni*.

**Abstract:**

Rumen flukes are geographically widespread trematodes affecting wild and domestic ruminants. The juvenile forms, which are found in the small intestine, are more pathogenic compared to the adults. Severe diarrhoea and weight loss are the major clinical signs, and the disease might be fatal in severely infested individuals. In the last decade, paramphistomosis has been described as an emerging parasitic disease in Europe. This study aimed to identify the rumen fluke species in wild ruminants from western Romania. Fifty-two pre-stomachs obtained from roe deer (*Capreolus capreolus*) that were hunted on 14 hunting grounds from Timiș and Arad counties were examined for the presence of paramphistomes. Three (9.09%) out of 33 samples were positive in Timiș County, and one (5.26%) out of 19 samples was positive in Arad County. Subsequent PCR testing revealed that three samples were positive for *Calicophoron daubneyi* and one for *Paramphistomum leydeni*. The presence of *C. daubneyi* and *P. leydeni* in roe deer has not been previously reported in Romania. Two *Paramphistomum* species—*C. daubneyi* and *P. leydeni*—were revealed as the main species of rumen flukes in roe deer from forests in Romania.

## 1. Introduction

Trematode infections, commonly referred to as “flukes”, are diseases occurring in sheep, goats, and cattle, but they can also affect other grazing animals as well as carnivores and birds. They are considered an animal health and welfare concern, impacting the livestock industry due to their influence on livestock production and output. The flukes are equipped with two suckers used to adhere to the host’s internal organs and used for feeding. Flukes cause harm to their hosts by feeding on blood, tissues, or other fluids and impairing the function of the affected organs through their mechanical action. This process leads to chronic wasting in animals [1]. The parasites mainly affect small and large ruminants like cattle, buffalo, deer, sheep, or goats [2]. Paramphistomosis is caused by ruminal parasites belonging to the Paramphistomatidae family that affect various species of domestic and wild ruminants. Rumen flukes are classified into six major genera with over 70 recognized species. Mature parasites are pink and pear-shaped, measuring around 0.5–1.0 cm in length. They attach firmly to the host rumen wall with a posterior muscular acetabulum, their gut is bifurcated, and they have an oral orifice which protrudes into the rumen. The immature forms are bright red and measure approximately 2–3 mm long.

The external morphology of the immature forms is very similar to the adult form. Their life cycle resembles the one in the liver fluke species, *Fasciola hepatica* [3]. Infection begins when the animals ingest metacercariae encysted on plants. At first, the juvenile forms of the parasite colonize the duodenum over a period of several weeks or months sometimes. The flukes are found attached to the mucosa with the help of their posterior suckers, and they do not affect layers deeper than the submucosa. After reaching the size of 1–3 mm, they begin their migration towards the rumen, where they become adults [3]. The difference between rumen fluke eggs and liver fluke eggs consists in the fact that the first are colourless. Eggs mature in the external environment and require temperatures of 25–27 °C. The next stage of the life cycle is the stage of ciliated miracidia, which hatch from the eggs and start searching for an intermediate host, the most common one being the mud snail *Galba truncatula* [4,5] both for the liver fluke and the rumen fluke. Due to this fact, there is competition between the two parasites once they enter the snail’s body. In the case of co-infection in an intermediate host, the first to reach the digestive glands, where access to nutrients is easier, will be the parasite that gets to develop faster to the next stage, namely the rediae [6]. However, this situation is very rare in the field, leading to the conclusion that this co-parasitism might be harmful to the survival of the intermediate host [6,7,8]. The cercariae emerge from the snails and encyst on vegetation, waiting to find the next host, which gets infected during grazing.

The emergence of rumen fluke in multiple European countries such as Spain [9], France [10], Belgium [11] and the UK [12,13] has been described by many researchers throughout the past decade. The increase in the incidence of this parasitic disease might be supported by several factors such as climatic changes, raised awareness on the diagnosis of this disease, and replacement of broad-spectrum anthelmintics with substances targeting specific parasites, especially the liver fluke, with little to no effect on the rumen fluke for example.

Literature states that *Calicophoron daubneyi* (syn. *Paramphistomum daubneyi*) is the predominant species in the western part of Europe [12,13,14,15], while in the eastern part of the continent, most studies report the presence of *Paramphistomum cervi*, in cattle, sheep and red deer, fallow deer or other wild ruminants [16,17]. *Paramphistomum leydeni* specifically affects wild ruminants, but it has also been identified in domestic goats from China [18], cattle from the Netherlands [19] and Argentina [20], sheep from Ireland [21] and reindeer from Finland [22]. There are reports on a recent importation of *C.daubneyi* in Ireland suggesting that the adaptation of this parasite to the intermediate host and climate conditions of the country might be a reason for the expansion of this disease [23]. In Romania, species such as *Paramphistomum cervi*, *P. daubneyi*, *P. microbothrium, P. gotoi* and *P. ichikawai* were identified over the years by means of morphology [24,25].

Clinical diagnosis is based on coproscopic detection by the sedimentation method [26]. Rumen fluke species used to be identified based on morphological characteristics; however, this method is often difficult to undertake correctly due to the increased risk of species confusion (some species were only differentiated after the implementation of molecular tools due to almost identical morphological features) [27,28]. Therefore, molecular assays are an efficient species identification and differentiation alternative, mostly using the ITS-2 gene fragment as a specific marker [29,30]. Useful studies in the classification and identification of these species of rumen flukes were carried out by Itagaki et al. (2003) [31] and Rinaldi et al. (2005) [15], who thus characterized several species of *Paramphistomum.* Control of rumen fluke is achieved with the help of anthelmintic drugs, mainly oxyclozanide, administered in two doses, three days apart, or similar efficacy has been demonstrated when using closantel [32].

A limited number of studies from western Romania focus on parasitism in both domestic and wild ruminants, and most of them focus more on other categories of parasites and less on trematodes [33,34,35,36,37,38,39,40]. Since information on the status of paramphistomosis in wild ruminants from Romania has not been recently updated [41,42], this study aimed to evaluate the presence and distribution of these parasites, using roe deer (*Capreolus capreolus*) as study subjects.

## 2. Materials and Methods

### 2.1. Sample Collection

**Figure 1:**
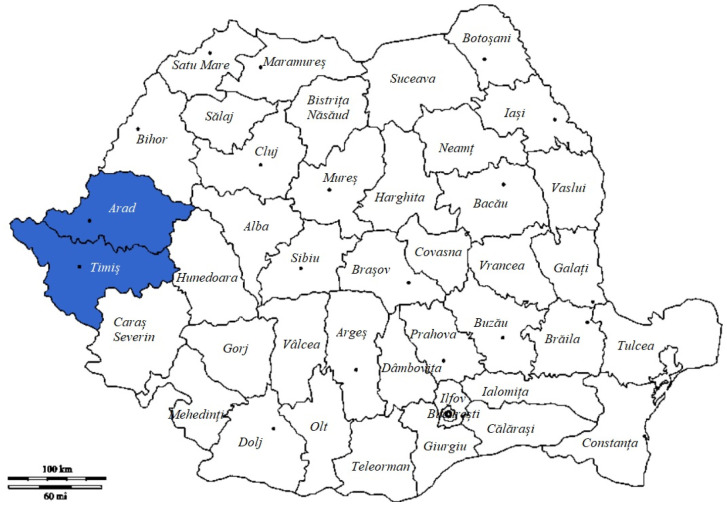
Sampling locations. Color: The areas marked with the color blue represent the studied geographical area.

The study was carried out in two counties situated in western Romania, namely Timiș and Arad (between 45°47′ N 21°21′ E and 46°22′ N 21°48′ E) (Figure 1), in the period 2019–2020. The collected samples consisted of roe deer pre-stomachs.

The samples came from 14 hunting grounds, namely, 19 from Arad County and 33 from Timis County (see Table 1 in the Result section).

**Figure 2:**
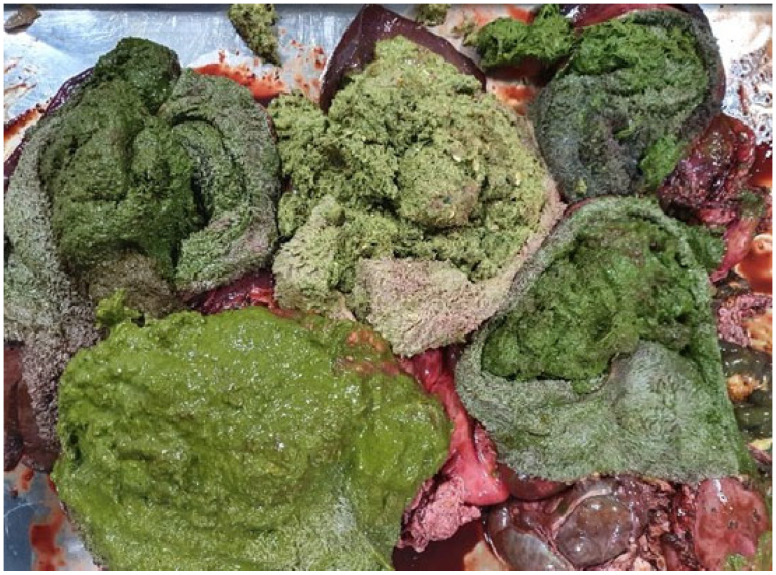
Several roe deer rumens prepared for examination.

**Figure 3:**
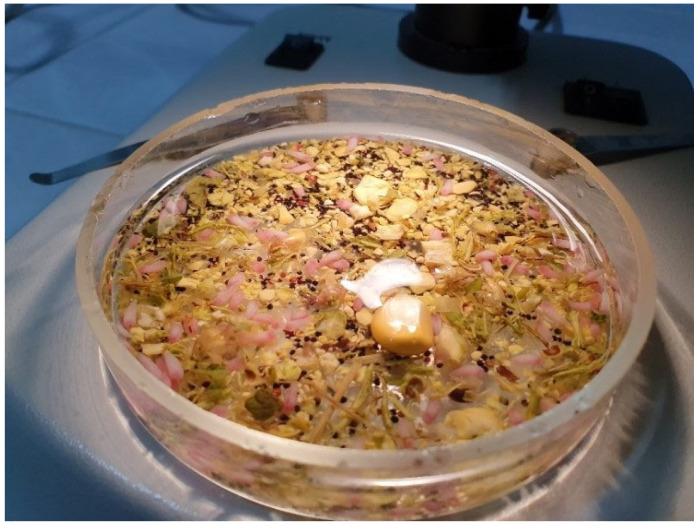
Sediment examination with the stereomicroscope; multiple rumen flukes are present.

All samples were preserved in portable refrigerating boxes and transported to the Faculty of Veterinary Medicine Timișoara, where they were further processed within the Parasitology Department Laboratories. The pre-stomachs were opened and washed with tap water to observe the presence of possible parasites (Figure 2). The stomach contents were examined using the sedimentation method, and the sediment was analysed with a stereomicroscope (Figure 3).

### 2.2. PCR Assay

Four pools of 5–10 adult paramphistomes were prepared for DNA extraction from the samples identified as positive. Extraction was performed using the BIOLINE^®^ kit (London, UK), according to the manufacturer’s instructions.

The PCR targeting the internal transcribed spacer 2 (ITS2) fragment was performed according to the technique described by Lotfy et al. (2010) [31], with some minor amendments required for the employed PCR mixture MyTaqTM Red Mix (BIOLINE^®^). The amplification itself was performed by classical PCR (manual PCR) and was based on the creation of several copies of a ~385 bp gene sequence for *Paramphistomum* spp.

The following primers were used: primer GA1: 5′-AGAACATCGACATCTTGAC-3′and revers BD2: 5′-TAT GCT TAA ATT CAG CGG GT-3′.

A Master Mix MyTaqTM Red Mix (BIOLINE^®^) was used to conduct the reaction. The final volume of the PCR reaction was 25 µL, of which 12.5 µL MyTaqTM Red Mix (BIOLINE^®^), 1 µL forward primer, 1 µL reverse primer (diluted to a concentration of 10 pmol/µL, according to the protocol described by the manufacturer), DNA extracted from the sample to be analyzed and ultrapure water.

The amplification program was performed with the thermocycler My Cycler (BioRad^®,^ Hercules, CA, USA). This program included the stages of DNA denaturation at 95 °C, for 1 min; 32 cycles of denaturation at 95 °C, 30 s; hybridization at 55 °C, 30 s; extension at 72 °C, 30 s; incubation at 4 °C.

The analysis and control of the amplicons were carried out by horizontal electrophoresis in a submerged electrophoresis system in 1.5% agarose gel, with the addition of the fluorescent dye MidoriGreen (Nippon Genetics^®^ Europe, Duren, Germany) at a voltage of 120 V and 90 mA, for 60 min. For the visualization and comparison of the results in the electrophoresis, a DNA Leader HyperLadder™ 100 bp (Bioline, London, UK) was used. The results were visualized in the UVP PhotoDoc-It (UVP^®^, Upland, CA, USA) imaging System with UV Transilluminator.

### 2.3. Sequencing

The PCR products were sequenced at Macrogen Europe^®^ Company (Amsterdam, The Netherlands) and compared with those available in the GenBank database using BLAST alignment (Table 2). Our sequences were uploaded in the GenBank database under the following accession numbers: OM953778.1 for *Paramphistomum leydeni* and OM913616.1 for *Calicophoron daubneyi*, respectively.

### 2.4. Phylogenetic Analysis

Additional sequences were searched in the National Center for Biotechnology Information (NCBI) Nucleotide repository. MAFFT [42] was used for multiple sequence alignment with default parameters for the all-sequence data of the ITS-2 fragments. The phylogeny of the selected strains was built by analyzing ITS-2 fragments using the R phangorn package [43] as follows. A distance matrix-based phylogenetic tree was constructed using the Neighbor-Joining (NJ) method. The Maximum Likelihood (ML) of the phylogenetic tree was estimated using the best-fit model. The Node support for this topology was calculated with 100 thorough bootstrap replicates. Data visualization was performed in the R ggtree package [44]. All additional data management steps were performed in an R environment (v4.2.2).

## 3. Results

In Timis County, three (9.09%) samples from a total of 33 samples collected from two hunting grounds turned out to be positive, while in Arad County, only one (5.26%) sample from only one hunting ground from a total of 19 examined samples turned out positive. Overall, rumen flukes were present in deer from three (21.42%) hunting grounds.

From a total of 52 examined samples collected from 14 hunting grounds located in Timiș and Arad counties, only four were positive for *Paramphistomum* spp. (Table 1), revealing an overall prevalence of 7.69%.

Three samples were found positive for *Calicophoron daubneyi*, and one was positive for *Paramphistomum leydeni* subsequent to PCR screening (Table 2).

**Figure 4:**
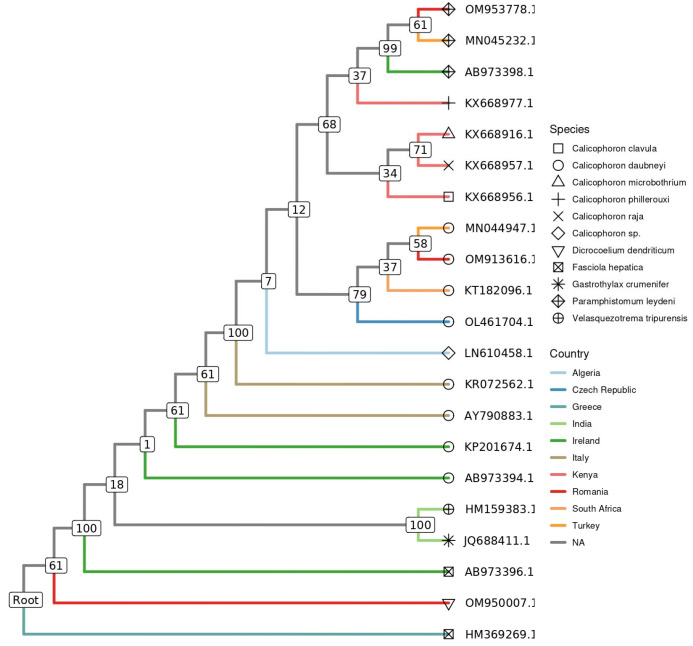
Phylogenetic tree showing the genetic relationship of the wildlife origin *Calicophoron daubneyi* (OM913616.1) and *Paramhistomum leydeni* (OM953778.1) sequences obtained in the present investigation and other paramphistomatids (marked with different geometric figures) isolated from different hosts, in different countries (marked with coloured lines), based on analysis of a partial sequence of the ITS2. GenBank^®^ accession numbers are shown, and the liver fluke, *Fasciola hepatica* (HM369269.1), was used as outgroup. Numbers at branches indicate bootstrap support levels (1000 replicates).

Phylogenetic analysis based on ITS2 gene sequences showed that the digenetic trematodes species, derived from the present investigation and included in the phylogenetic tree construction, clustered closely with those selected and downloaded from the GenBank^®^ database. Thus, our *P. leydeni* (accession no. OM953778.1) and *C. daubneyi* (accession no. OM913616.1) isolates branched separately, into one of the two resulting main clades, together with other sequences isolated from sheep (*Ovis aries*) (accession no. MN045232.1) and cattle (*Bos taurus*) (accession no. 044947.1) in Turkey, respectively (Figure 4)

## 4. Discussion

It is a well-known fact that deer have been considered potential reservoirs for pathogenic agents such as TBC or the blue-tongue virus that also affect other animals, both domestic and wild [45,46]. Thus, wild ruminants may or may not carry the same *Paramphistomum* species as domestic ruminants in certain regions. In this regard, no rumen flukes were found in over 200 deer killed in Galicia (Spain) near several cattle ranches. However, *C. daubneyi* was found in cattle [9]. In the Republic of Ireland, cattle and wild ruminants (except for the Sika deer) were parasitized by *C. daubneyi*, while *P. leydeni* was diagnosed only in the cervids [47]. A study from Finland reported *P. leydeni* as the main species affecting reindeer, with seasonal variations in rumen fluke morphology. The study reported that the parasites collected in winter were smaller and immature than those collected in summer, which were the mature parasite stage [22]. The infection, which was thought to be limited to the tropical and subtropical regions, has been increasingly reported in temperate countries. Thus a study from Croatia reported concurrent infection with *P. leydeni* and *P. cervi* in a red deer doe, making this the first report of *P. leydeni* in Croatia [19]. Authors from Great Britain performed DNA sequencing of the ITS-2 region to establish the species of rumen fluke present throughout the country. Despite previous reports stating that *P. cervi* was the most common rumen fluke found throughout the UK in cattle and sheep, the DNA test revealed *C. daubneyi* to be the most common one [12]. Molecular and morphological characterization of *Paramphistomum* spp. samples collected from cattle in Argentina demonstrated that in this country, where the disease is considered an emerging one, impacting an increasing number of cattle herds, the predominant species is *P. leydeni* [48]. Regarding other wild animals, water buffaloes from Turkey were predominantly parasitized by *C. daubneyi* and at lower rates by *P. cervi* [49]. Other species of paramphistomes, namely *P. microbothrium* and *P. cervi*, were identified in wild ruminants from Slovakia [50] and Serbia [51], countries located in the vicinity of Romania.

*C. daubneyi* is considered the most widespread species of rumen fluke throughout Europe, especially in cattle [12,52]. The adult forms induce inflammatory changes in the rumen and reticulum; however, it is considered that this life cycle stage is of low clinical significance [53]. Its young forms, namely the metacercariae, which are found in the intestinal mucosa, are responsible for clinical manifestations such as diarrhoea and weight loss, which can lead to the death of affected individuals in case of severe infection such as that described by O’Shaughnessy et al. (2018) [23] which reported a severe rumen fluke outbreak in 6-month-old heifers from Ireland. Another study from Ireland reported a spike in deaths due to paramphistomosis in a certain year when rainfall was heavier than usual, demonstrating that the high burdens of juvenile parasites were responsible for the occurrence of the severe forms of the disease [16]. In cattle, the frequency varies by country and reporting technique (laboratory testing or identification in the slaughterhouse), but it is higher in Western Europe: 32–52% in Ireland, among the highest ones throughout Europe, identified using PCR amplification and DNA sequencing of a ~500 bp fragment of ITS-2 [49], 6–61% in Spain where identification was carried out using both morpho-anatomical and molecular techniques confirming the presence of *C.daubneyi* [9], 45% in France [10], 22–28% in Belgium [52], except for Germany, where the incidence is of only 5.5% [53]. Infections with *C. daubneyi* were also confirmed in the Czech Republic, where one study in particular also found positivity among animals housed in stables, evidence suggesting the possibility of transmission of this parasite through roughage [54].

On the other hand, the information regarding the spread of *P. leydeni* is controversial. Several studies aimed to determine the best method to identify and diagnose the presence of this parasite in animals by comparing several methods, such as the performance of the mini-FLOTAC used to identify adult rumen fluke infections through the faecal egg count on animals from a slaughterhouse or the association between herd-diarrhoea problems and the presence of rumen fluke. The FLOTAC method proved highly sensitive and specific; however, no connection was noticed between the presence of diarrhea and that of the fluke [11]. In most cases, the identification of paramphistome species was based on morphological characteristics, data that did not always prove useful, due to frequent confusion of *P. leydeni* with *P. cervi* or *P. epiclitum* [10,17,55].

Accurate species identification of rumen flukes carries great utility for the proper investigation of the epidemiology and pathophysiology and also for the economic impact that these species exert [55]. The introduction of molecular biology tests in Europe in the 2000s [50,56,57,58] considerably reduced the possibility of inherent confusions in the case of morphological examinations, the diagnoses having a high accuracy, even allowing the identification of several species of paramphistomes within the same herd or in different herds, located in each other’s proximity. Thus, in Germany, animals from 34 farms were screened using PCR, of which 24 proved positive for *C. daubneyi*, and 4 for *P. leydeni* [26]. The species-specific ribosomal internal transcribed spacer 2 (ITS2) markers were designed for PCR differentiation between liver fluke species and rumen species [50]. Various methods of DNA isolation were studied, with findings reporting that the best method of detecting and molecularly identifying *C. daubneyi* was the method based on phenol-chloroform purification, with supporting evidence for the utility of the ITS-2 fragment, the one we also used in the present study, as a genetic marker for the molecular identification of *C. daubneyi* in both definitive and intermediate hosts [57].

The interactions between the different species of paramphistomes are feasible because, on the one hand, both species of paramphistomes can share the same intermediate host, but also because grazing areas for domestic and wild ruminants can overlap. A study from Germany focused on the distribution of rumen flukes and reported differences in the geographical distribution of rumen and liver flukes, with a higher prevalence of rumen flukes in the northern part of Germany. The study also concluded that the animals sharing pasture with other ruminants had a higher risk of being infected with rumen flukes [59]. These factors also apply to the western region of Romania, where this study was conducted.

In the Netherlands, Ploeger et al. [21] conducted a study on ruminal trematodes in cattle and sheep over six years from 2009 to 2014. The usual coproscopic examinations (sedimentation methods) revealed a prevalence of 8% for sheep and 15.8% for cattle. The investigation was added using the modified Dorsman quantitative method, which had a sensitivity of 95% for weak positive samples, a method validated by Dutch Animal Health in Deventer (GD Deventer). Of the paramphistoma samples collected from slaughterhouses, 14 were subjected to PCR analysis using primers ITS-2F: 5′-TGTGTCGATGAAGAGCAG-3′ and ITS-2 R: 5′-TGGTTAGTTTCTTTTCCTCCGA-3′, recommended by Itagaki et al. (2003) [31] and Rinaldi et al. (2005) [12]. Thus, after comparing the sequences with those in Genbank, 12 samples were positive for *C. daubneyi* (eight from cattle and four from sheep), and the other two, also from cattle, were identified as *P. leydeni*. Both sequences matched 99.8–100%.

The structure of the resultant phylogenetic tree highlighted that our isolates were grouped in distinct clades, together with other representative GenBank-deposited paramphistomatid sequences, isolated from various hosts in different countries and from several geographical regions of the world. This phenomenon has been previously described by Mitchell et al. [58]. The evolutionary distance analysis indicates an affined bootstrap support level between *P. leydeni* and *C. daubneyi*. The observed discrimination between these different non-host-specific digenetic trematodes suggests that the used genetic markers are reliable and representative in supporting the presence of genetic differences between the evaluated species infecting wild ruminants.

The role of deer as reservoirs was also studied by O’Toole et al. (2014) [60] in Ireland. Commonly recognized as reservoirs for disease in livestock, the fallow deer, red deer, and sika deer also proved to be carriers of rumen fluke. The study reports fallow deer to be frequently infected with two rumen fluke species, *C. daubneyi* and *P. leydeni*, similar to the result found in our study in deer from Romania. The amount of *C. daubneyi* eggs shed by fallow deer was significant in the study conducted in Ireland. However, the authors do not consider them a risk factor for infection in cattle. The parasites were detected in red deer as well, but at a lower rate.

A survey conducted on cattle and deer from The French archipelago New Caledonia revealed high prevalence rates, up to 70% in cattle and 47% in deer, for rumen flukes. The species reported in this study, *Calicophoron calicophorum*, *Fischoederius elongates,* and *Orthocoelium streptocoelium*, were identified using ITS-2 sequencing. The fact that all three species were present in both cattle and deer suggests the possibility of rumen fluke transmission between the two host species, supporting the idea of parasite transmission at the livestock-wildlife interface [61].

Studies on rumen fluke in wild animals continue to be scarce compared to those focusing on livestock, and there is little information available on the possible implication of rumen fluke from deer in the pathogenesis of cattle that graze on the same or nearby pastures despite the obvious evidence supporting the possibility of interspecific transmission.

## 5. Conclusions

The present study, conducted in two counties found at the border of Romania with both Serbia and Hungary, revealed the existence and complementarity of two *Paramphistomum* species—*C. daubneyi* and *P. leydeni*—in wild ruminants, more specifically in roe deer. This is the first study in Romania to use molecular methods to identify rumen fluke species.

## Figures and Tables

**Table 1:** Screening results of samples collected from all 14 hunting grounds.

Crt.No	County	Hunting Ground	Total Samples	Positive Samples (%)	Negative Samples (%)
1.	Timiș	Bethausen	9	2 (22.22%)	7 (77.78%)
		Valea Lungă	4	-	4
		Făget	1	-	1
		Ohaba	7	1 (14.28%)	6 (85.72%)
		Surduc	4	-	4
		Traian Vuia	5	-	5
		Nevrincea	3	-	3
2.	Arad	Săvârșin	5	1 (20%)	4 (80%)
		Dumbrava	3	-	3
		Moneasa	1	-	1
		Lipova	3	-	3
		Petriș	2	-	2
		Șimand	4	-	4
		Fiac	1	-	1
TOTAL			52	4	48

**Table 2:** Results of PCR sequencing.

Sample No.	Sample Identification	Codon		Similar Sequence	Species
		Start	End		
1.	254	10	1310	KJ995531.1	*Paramphistomum leydeni*
				KJ995529.1	
				AB973398.1	
				HM209064.1	
2.	255	10	1040	KP201674.1	*Calicophoron daubneyi*
				AY790883.1	
3.	256	10	920	AB973394.1	*Calicophoron daubneyi*
4.	259	10	850	KT182100.1	*Calicophoron daubneyi*
				KT182096.1	

## Data Availability

No new data were created or analyzed in this study. Data sharing is not applicable to this article.
